# Effects of Poly (ADP-ribose) Polymerase Inhibition on DNA Integrity and Gene Expression in Ovarian Follicular Cells in Mice with Endotoxemia

**DOI:** 10.52547/ibj.26.1.44

**Published:** 2021-11-27

**Authors:** Olena Kondratska, Nataliya Grushka, Svitlana Pavlovych, Nataliya Krasutska, Serhii Tsyhankov, Roman Yanchii

**Affiliations:** 1 Bogomoletz str, Kyiv, Ukraine, 01024- Department of Immunophysiology, Bogomoletz Institute of Physiology, NAS of Ukraine;; 2 Grafska str, Nizhyn, Ukraine, 16600- Department of Chemistry and Pharmacy, Nizhyn Mykola Gogol State University, Ukraine

**Keywords:** Cell death, Gene Expression, Lipopolysaccharides, Oocytes, Poly (ADP-ribose) polymerase-1

## Abstract

**Background:**

A mouse model of LPS-induced inflammation was used to investigate the effect of pharmacological inhibition of nuclear enzyme PARP-1 on oocyte maturation, apoptotic and necrotic death, as well as DNA integrity of follicular cells. Also, the relative expression of cumulus genes (*HAS2*, *COX2*, and *GREM1*) associated with oocyte developmental competence was assessed.

**Methods:**

Mice were treated with the PARP-1 inhibitor, 4-HQN, one hour before LPS administration. After 24 h, oocyte *in vitro* maturation was detected. Granulosa cell DNA damage was determined by the alkaline comet assay. Live, necrotic and apoptotic cells were identified using double vital staining by fluorescent dyes, Hoechst 33342 and propidium iodide. The expression levels of cumulus genes were assessed using reverse transcriptase PCR.

**Results:**

The administration of 4-HQN to LPS-treated mice ameliorated oocyte meiotic maturation and exerted a significant cytoprotective effect. 4-HQN attenuated LPS-induced DNA damage and favored cell survival by decreasing necrosis and apoptosis in granulosa cells. Exposure to 4-HQN increased mRNA expression levels for *HAS2*, *COX2*, and *GREM1* in cumulus cells.

**Conclusion:**

The obtained results indicate the involvement of PARP-1 in the pathogenesis of ovarian dysfunction caused by LPS. We suppose that this enzyme can be an attractive target for the therapy of inflammatory disorders in ovary. The protective action of PARP-1 inhibition could at least partly be associated with the reduction of necrotic death of follicular cells and also in other cells. However, the detailed mechanisms of the *favorable effect* of PARP inhibitors on endotoxin-induced ovarian disorders need to be further explored.

## INTRODUCTION

Afemale genital tract infection with Gram-negative bacteria can disturb normal ovarian function and result in infertility^[^^1^^-^^3^^]^. The lipopolysaccharide endotoxin is an important surface membrane component in these bacteria. It has been shown that LPS can induce ovarian pathology by affecting the functions of follicular cells and oocyte developmental competence*.* Mice treated with the endotoxin had decreased a number of primordial follicles. Also, LPS inhibited estradiol production in granulosa cells and progesterone production in theca cells, representing an endocrine-disrupting effect^[^^1^^,^^4^^,^^5^^]^. This endotoxin disrupted meiotic progression, mitochondrial distribution in the cytoplasm, and mitochondrial membrane potential, which caused the disruption of nuclear maturation of bovine oocytes^[6]^. Furthermore, LPS exposure exhibited increased reactive oxygen species levels, enhanced apoptotic gene expression, and changed epigenetic status in bovine oocytes^[^^7^^]^. It is important to note that the LPS level in the follicular fluid, which surrounds and nourishes oocytes, is close to those in circulating blood. Therefore, it is obvious that systemic endotoxemia can be related to ovarian inflammation^[^^1^^,^^8^^]^. 

PARP-1 is a nuclear enzyme essential for various cellular functions, including DNA damage detection and repair, transcriptional regulation, and cell death. It belongs to a family of 18 enzymes that *utilize* NAD^+^ as a substrate to form large negatively charged polymers of poly (ADP-ribose) and attach them to acceptor proteins, thereby modifying their function. PARP-1 is activated upon binding to DNA strand break and initiates the repair of damaged DNA and preservation of genomic integrity^[^^9^^,^^10^^]^. Therefore, optimal expression and activity of this enzyme are necessary for a variety of cellular processes (e.g. transcriptional regulation, chromatin modification, cell proliferation, and death); however, overactivation of PARP-1 can contribute to tissue damage and inflammatory disorders. It has been shown that proinflammatory cytokines (TNF-α and IL-1), free radicals, and bacterial products (such as LPS) can activate PARP-1^[^^10^^,^^11^^]^. This enzyme participates in the pathogenesis of various immune-mediated diseases, comprising rheumatoid arthritis, autoimmune nephritis, and atherosclerosis, mainly through the activation of proinflammatory transcription factors (nuclear factor kappa B and activating protein-1) and the increase in a necrotic type of cell death^[^^12^^,^^13^^]^. It has been displayed that in PARP-1 knockout mice that inflammation and tissue damage reduced under various pathological conditions, inhibitors of the enzyme have been reported to have similar beneficial properties^[^^9^^,^^14^^,^^15^^]^. It is expected that the inhibitors of PARP-1 can be a promising tool for therapeutic intervention^[^^16^^-^^19^^]^. It also can be assumed that the inhibition of this nuclear enzyme could have a protective effect on the disorders of the female reproductive system associated with endotoxemia. In this respect, the effect of PARP-1 inhibitor, 4-HQN^[^^19^^]^, on oocyte meiotic maturation, apoptotic and necrotic death, and also DNA integrity of granulosa cells in mice with LPS-induced endotoxemia was studied. Changes in the expression of genes, i.e. *HAS2*, *COX2*, and *GREM1*, which may serve as markers of oocyte quality, were also investigated in similar conditions. 

## MATERIALS AND METHODS

**Animals**

The study was conducted with adult female Albino mice (18-20 g; 6-8 weeks of age; Experimental Biological Clinic of Bogomoletz Institute of Physiology, Ukraine). The mice were placed into the cages (four per cage), and each was individually ventilated with 12-hour light/dark cycle, maintained at 22 ± 2 ºC. All the mice were provided with certified rodent diet and filtered water *ad libitum*.

**E** **xperimental design**

The estrous cycle stages were identified by vaginal smears. Female mice in both metestrus and diestrus phases were randomly divided into four groups (eight mice per control and experimental group): (1) mice treated i.p. with vehicle-saline (control group); (2) mice treated i.p. with 3 mg/kg of LPS (*E. coli* 0111:B4, Sigma-Aldrich, St. Louis, MO, USA); (3) mice received an injection of 4-HQN (100 mg/kg; i.p.; Sigma-Aldrich); (4) mice treated with 4-HQN, 1 h before LPS challenge. Twenty four hours after LPS administration, ether anesthesia was used for *euthanizing* mice, and then murine ovaries were sampled. 

**Determination of oocyte meiotic maturation**

The large antral follicles with four or more layers of granulosa cells were isolated from ovaries using light microscopy. Cumulus oocyte complexes were separated mechanically and cultured in DMEM (Sigma-Aldrich) at 37 °C, supplemented with 5% fetal bovine serum and the antibiotics penicillin (100 U/ml) and streptomycin (100 µg/ml) (Sigma-Aldrich). The number of oocytes at metaphase I stage (with germinal vesicle breakdown) was calculated by light microscopy after 4 h of cultivation; the number of metaphase II oocytes (with the first polar body) was counted after 20 h of cultivation. The oocyte maturation rate was calculated by using the ratio of total metaphase I oocytes and metaphase II oocytes to the total oocyte number in the group. 

**Determination of the cell death**

Freshly isolated cells were used for quantitative evaluation of viability and death. Follicular (granulosa) cells were obtained after oocyte removal from cumulus oocyte complexes and dispersion by careful pipetting. To define the percentage of live, necrotic and apoptotic cells, we stained the cells with fluorescent dyes, propidium iodide, and Hoechst 33342^[^^20^^]^. Because propidium iodide penetrates the damaged plasma membranes and stains the cell nuclei in red, only necrotic cells emit red fluorescence. Hoechst 33342 *enters* live cells with intact membranes, staining their nuclei in blue. The binding of these dyes to chromatin allows identifying the nuclear apoptotic features, such as chromatin condensation, DNA fragmentation, and apoptotic body formation. Staining was performed in PBS with a final concentration of 10 μmol/l for each dye. The cells were then kept in darkness for 10 minutes and subsequently washed with PBS by centrifugation. In the next step, cells were fixed in 5% formalin in PBS for two minutes, followed by repeated washing. Smears were prepared and studied under a fluorescent microscope (×700). In each sample, at least 100 cells were counted, and the percentage of live, necrotic and apoptotic cells was determined. 

**С** **omet assay**

To assess DNA damage in granulosa cells, the alkaline comet assay procedure was performed as described earlier^[^^2^^]^. Briefly, the alkaline single cell gel electrophoresis assay detected DNA strand breaks, alkali labile sites, and incomplete excision repair sites. The comets were detected and scored by visual inspection according to Collins^[^^21^^]^, and the TCS was evaluated.

Determination of gene expression

Total RNA extraction from cumulus cells was performed using Trizol RNA Prep 100 kit (Isogen, Russian) according to the manufacturer’s instruction. Reverse transcription was carried out using the First Strand cDNA Synthesis Kit (Fermentas, Lithuania) as described in the manufacturer’s protocol. Total RNA samples (5 μl) were used as templates. After thawing, all components were mixed by brief vortexing and then placed on ice. In the next step, RNA template (5 μl), Random Hexamer primer (1 μl; 0.5 μg/μl), and nuclease-free water (6 μl; to reach the final volume of 12 μl) were added to a sterile, nuclease free thin-walled microcentrifuge tube (0.2 ml), prechilled on ice. The reaction mixtures were prepared for both the positive and negative controls without the template. The contents were gently vortexed and incubated at 70 °С for 5 minutes*.* After incubating, the contents of tubes were placed on ice. Afterward, 5× RT Buffer (4.0 μl), 2 μl of dNTP mix (10 mM each), 0.5 μl of RiboLock RNAse inhibitor (40 U), 1.5 μl of M-MuLV Reverse Trascriptase (20 U/μl) were added to make a total volume of 20 μl; all reagents were purchased from Fermentas). The contents of tubes were gently vortexed. Then reverse transcription of RNA into cDNA was performed by incubating at 37 °С for 120 minutes. The final stage of the reaction process was heating at 70 °С for 10 minutes. Contents were placed on ice. Next, single strand cDNA obtained was used for PCR (Applied Biosystems 2700, PerkinElmer, USA), which performed using specific primers for each gene. GAPDH was *applied* as a housekeeping gene for normalizing PCR results. The list of PCR primers are presented in Table 1. PCR products were separated using agarose gel electrophoresis and visualized using a UV-transilluminator (Biokom, Russian). The fluorescence intensiveness was assessed by ViTran program (version 1.00 for Windows, Biokom, Russian).

**Statistical analysis**

The GraphPad Prism *software* version 5.00 for Windows (San Diego, California, USA) was used for statistical analyses. The normality of the data distribution was analyzed by Kolmogorov-Smirnov test. In the case of normal data distribution, one-way ANOVA with Newman Keuls post hoc test was applied. The results were expressed as mean ± SEM. Kruskal-Wallis test and Dunn's multiple-comparison test were applied for data with non-normal *distribution*. * p *values less than 0.05 were considered statistically significant. 

**Table 1:** List of PCR primers used for experiments and PCR product size

**Gene**	**Sequence of primers **	**Product size (bp)**
*HAS2*	F: 5'-CCTCCAGTTAGTGTCTGGGTTC-3' R: 5'-CTGTGCAGCTATTCCTGTGTTC-3'	409
		
*COX2*	F: 5'-GAAGGAACTCAGCACTGCATC-3' R: 5'-CAGTCCGGGTACAGTCACACT-3'	213
		
*GREM1*	F: 5'-AAGGCACTTCCTGTTACTCTGC-3' R: 5'-TACGACTGAGATGTCAGGGAGA-3'	256
		
*GAPDH*	F: 5'-GGGTGTGAACCACGAGAAATATGA-3’ R: 5'-AGCACCAGTGGATGCAGGGATGAT-3’	240

F, forward; R, reverse

**Ethical statement**

The above-mentioned treatment and sampling protocols were approved by the Biomedical Ethics Committee of Bogomoletz Institute of Physiology (Kyiv, Ukraine) and performed in accordance with the rules established by the Law of Ukraine No. 3447-IV "On protection of animals from cruelty", as well as the guidelines established by the EU Directive 2010/63/EU for animal experiments. 

## RESULTS

**Effect of** **PARP-1 inhibition on o** **ocyte meiotic maturation** **in mice with endotoxemia**

**Fig. 1:**
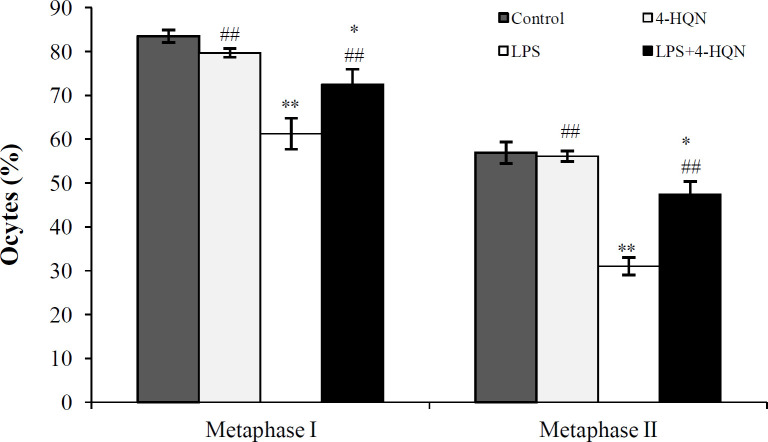
Effect of 4-HQN on the percentage of oocytes with germinal vesicle breakdown (metaphase I) and oocytes forming the first polar body (metaphase II) in mice treated with LPS. Control mice received saline. Results are expressed as mean ± SEM. ^*^p < 0.05 and ^**^p < 0.01 compared to saline controls; ^##^*p* < 0.01 compared to LPS-treatment

Our data indicated that under the condition of LPS-induced impairment of ovarian function, PARP-1 inhibition significantly increased the number of oocytes reaching metaphase I (with germinal vesicle breakdown) and II (with extruded the first polar body) compared to LPS group, indicating an improvement in their developmental competence (Fig. 1). Of note, 4-HQN treatment alone did not have an impact on oocyte meiotic maturation of intact mice (*p *> 0.05). 

**Effect of** **PARP-1 inhibition on** **gene e** **xpression in cumulus cells** **of mice with endotoxemia**

**Fig. 2:**
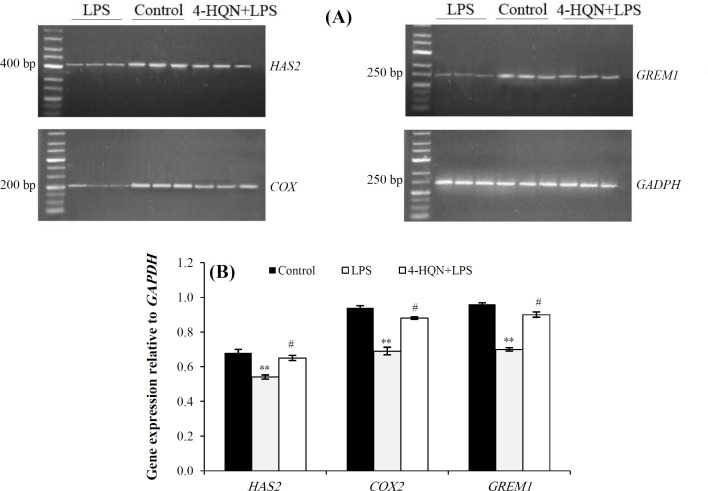
Effect of 4-HQN administration on *HAS2*, *COX2*, and *GREM1* gene expression in cumulus cells of mice treated with LPS. (A) Agarose gel electrophoresis of PCR products generated by specific primers for *HAS2*, *COX2*, and *GREM1*. (B) Relative expression ratio (bar graph) of *HAS2*, *COX2*, and *GREM1* normalized to *GAPDH* gene control. Data are represented as mean ± SEM. ^**^p < 0.01 compared to saline controls; ^#^*p* < 0.05 compared to LPS-treatment

The pretreatment with 4-HQN enhanced the expression of mRNA for all studied genes in cumulus cells obtained from mice with LPS-induced endotoxemia. The levels of *HAS2* mRNA expression increased by 20%, *COX2* by 28%, and *GREM1* by 29% (*p* < 0.05 for all) compared to LPS group. Expression levels of *HAS2*, *COX2*, and *GREM1* mRNA were detected in all of the samples (Fig. 2A). The relative expression profile of the genes is presented in Figure 2B. 

**Effect of** **PARP-1 inhibition on overall genome integrity in granulosa cells of mice with endotoxemia**

**Fig. 3:**
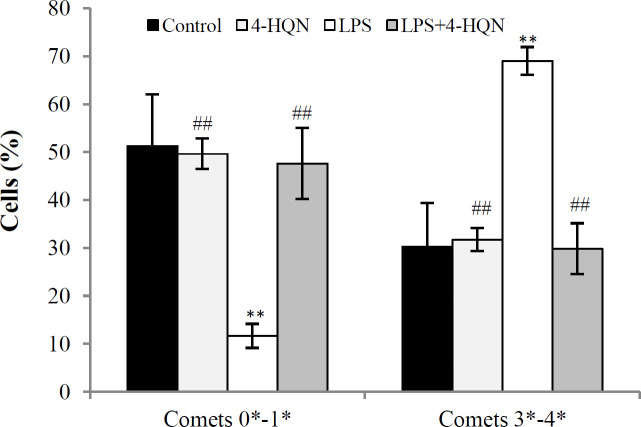
Effect of 4-HQN on the percentage of granulosa cells with undamaged and slightly damaged DNA (comets within classes 0 and 1) and cells with severe DNA damage (comets within classes 3 and 4) in mice treated with LPS. Control mice received saline. Results are expressed as mean ± SEM. ^**^p < 0.01 compared to saline controls; ^##^*p* < 0.01 compared to LPS-treatment

Endotoxemia caused a 1.9-fold elevation of TCS values (a cumulative index that considers the changes in the number of comets of each type with varying degree of DNA damage) in ovarian granulosa cells (from 156 ± 33 in control to 295 ± 9 in LPS group, *p* < 0.001). Also, LPS treatment led to a 2.3-fold increase (compared to the control) in the number of granulosa cells with severe DNA damage. The administration of PARP-1 inhibitor decreased TCS by 1.8-fold (from 295 ± 9 in LPS group to 167 ± 21 in 4-HQN + LPS group; *p* < 0.01) and reduced the number of granulosa cells with severe DNA damage nearly to the control levels (Fig. 3). 4-HQN, used *alone*, had no significant *effect* on the overall genome integrity in granulosa cells of intact mice (*p >* 0.05). The comet assay also indicated that PARP inhibition significantly attenuated endotoxin-induced genotoxicity in ovarian granulosa cells.

**Effect of** **PARP-1 inhibition on granulosa cell viability in mice with endotoxemia**

**Fig. 4:**
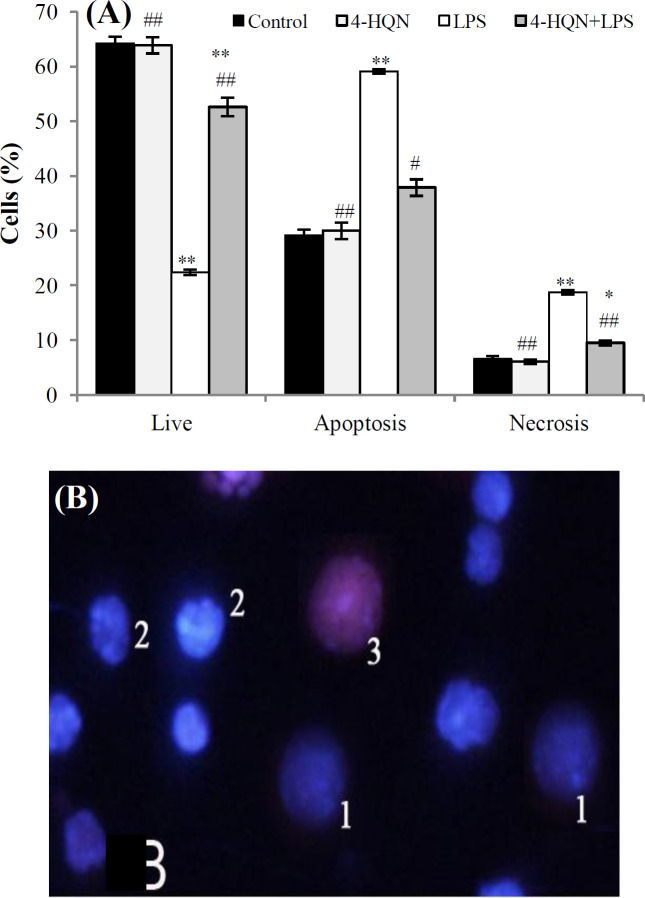
Effect of 4-HQN on the percentage of viable, apoptotic or necrotic granulosa cells on mice treated with LPS. Control mice received saline. (A) The percentage of viable, apoptotic and necrotic cells as determined by their vital staining with fluorescent dyes, Hoechst 33342 and propidium iodide; (B) representative image of cells stained by Hoechst 33342 and propidium iodide: 1, viable cells (Hoechst 33342 penetrates non-injured membranes and stains the nuclei of live cells in blue); 2, apoptotic cells (with characteristic nuclear changes: peripheral localization of chromatin, chromatin condensation, and nuclear fragmentation); 3, necrotic cells (propidium iodide penetrates through leaky plasma membranes and stains their nuclei in red). Data are represented as mean ± SEM. ^*^p < 0.05 and ^**^p < 0.01 compared to saline controls; ^#^*p* < 0.05 and ^##^*p* < 0.01 compared to LPS-treatment

It is known that a severe DNA injury can lead to different types of cell death, including proinflammatory necrotic death that can enhance inflammation and ovarian injury. In this study, LPS caused a pronounced decline in granulosa cell viability and an increase in the number of necrotic and apoptotic cells (*p* < 0.001; compared to control; Fig. 4A). Representative image of live, apoptotic and necrotic cell nuclei is presented in Figure 4B. The pretreatment with 4-HQN favored cell survival by increasing the amount of viable cells (*p* < 0.001 compared to LPS group) and decreasing the percentage of necrotic (*p* < 0.01) and apoptotic (*p* < 0.05) granulosa cells (Fig. 4A). 4-HQN *alone* did not have any *effect* on granulosa cell viability of intact mice (*p *> 0.05).

## DISCUSSION

LPS is widely used to establish mammalian models of immune-mediated inflammation. Earlier, we have demonstrated that intraperitoneal administration of LPS caused systemic inflammation in female mice. We also observed ovarian dysfunction, impaired oocyte meiotic maturation, strong genotoxic stress of ovarian follicular cells, elevated level of DNA damage in granulosa cells, and the changes in the mRNA level of certain cumulus genes, which are associated with oocyte developmental competence^[^^2^^]^. In the present study, we found that LPS *exposure* significantly decreased the viability of granulosa cells and increased the number of cells dying through the pro-inflammatory and immunogenic necrotic pathway. During systemic inflammation, it has been suggested that cumulus cells can initiate an inflammatory response to endotoxin because these cells express TLR4^[^^22^^]^. The mechanisms by which LPS negatively affect *ovarian function* are not yet completely understood. 

PARP-1 has been demonstrated to be involved in the regulation and maintenance of tissue inflammation^[^^11^^,^^23^^,^^24^^]^. Moreover, LPS increased the levels of PARP-1 mRNA^[^^25^^]^. As reported before, LPS was able to increase PARP-1 expression and activation by inducing DNA damage^[^^26^^]^. In our work, we showed that the administration of 4-HQN to LPS-treated mice had anti-inflammatory and cytoprotective effects. Therefore, the reduction of genotoxic stress and necrotic death of thymus and lymph node cells, as well as the significant decrease in functional and metabolic activity of neutrophils were revealed under these conditions^[^^27^^]^. Although the use of PARP-1 inhibitors can have the efficacy for the treatment of an inflammatory-induced tissue injury^[^^14^^,^^18^^,^^28^^,^^29^^]^, the inhibitory effects of this enzyme on LPS-induced ovarian dysfunction remain unclear. 

During the present study, the effect of PARP-1 inhibitor 4-HQN on the changes of ovarian function in mice with endotoxemia was examined. It was established that 4-HQN treatment resulted in an improved morphofunctional status of granulosa cells and an ameliorated oocyte meiotic maturation. During the folliculogenesis, several cumulus expressed genes are crucial for oocyte maturation and development^[^^30^^-^^32^^]^. We investigated the expression of three cumulus genes, *HAS2*, *COX2* (or *PTGS2*), and *GREM1*, which were previously reported in many papers and correlated with the high quality oocyte development^[^^31^^,^^33^^-^^35^^]^. Endotoxemia led to a significant decrease in the level of mRNA expression of *HAS2*, *COX2* and *GREM1* genes in cumulus cells^[^^2^^]^. However, 4-HQN administration to LPS-injected mice significantly increased the expression of these genes in cumulus cells surrounding oocytes. The obtained results indicated that the different expression pattern of the target genes can be applied as potential biological markers for the developmental competence of oocytes in the presence of LPS-induced pathological process. *HAS2* mRNA is a *necessary component* required for cumulus cell expansion, which is essential for oocyte maturation and ovulation process. During cumulus expansion, *HAS2* gene expression is involved in the synthesis of hyaluronic acid, one of the *main *components of the extracellular matrix^[^^36^^]^. *COX2* gene encodes the corresponding enzyme, which is involved in prostaglandin biosynthesis. *COX2* produced by cumulus cells covers an important role in cumulus expansion and meiotic resumption during oocyte development^[^^32^^,^^37^^]^. The involvement of *GREM1* in ovarian function is not *entirely clear*. It is known that *GREM1* is a BMP antagonist involved in the regulation of embryonic development. It has also been suggested that the selective inhibition of signaling pathways associated with BMP can direct growth differentiation factor 9 toward cumulus expansion during ovulation^[^^38^^]^. The mRNA expression of *GREM1* and *HAS2* has been found to be significantly lower in immature oocytes compared with mature cells^[^^39^^]^. 

Other authors have revealed a positive correlation of *PTGS2* with oocyte nuclear maturation^[^^32^^]^. The prominent percentage of studies has reported that the expression levels of *HAS2*, *GREM1*, and *COX2* were higher in cumulus cells separated from oocytes, which developed into high-quality embryos^[^^31^^,^^33^^-^^35^^,^^39^^]^. Therefore, the collecting data from this experiment, along with other published results, provides the rationale for assessing the expression of studied genes as biomarkers of oocyte quality. 

During infections, bacterial LPS is able to enter the bloodstream and to spread far from the site of infection^[^^40^^]^. In this regard, the presence of endotoxin has been documented in blood plasma and in follicular fluid^[^^40^^,^^41^^]^. As mentioned above^[^^22^^]^, cumulus and granulosa cells express TLR4 receptors; therefore, they have the potential to initiate an inflammatory response to LPS by increasing the expression of proinflammatory mediators (e.g. TNFα, IL-1β, IL-6, and IL-8)^[^^22^^,^^40^^,^^41^^]^. We hypothesize that LPS-induced inflammation in the follicular fluid impacts the cumulus-oocyte complex, and exposure to high levels of proinflammatory cytokines can have an adverse effect on cumulus cell signaling and disrupt the expression of studied genes. In particular, it has been demonstrated a decrease in the expression of *GREM1* in cumulus cells in women, under the influence of high levels of IL-1β and IL-10^[^^38^^]^. Therefore, the favorable effect of PARP-1 inhibition on endotoxin-induced ovarian disorders could be mediated by changes in the activation of proinflammatory transcription factors and intracellular signaling pathways as has been demonstrated in different models of inflammatory diseases^[^^9^^,^^19^^,^^42^^]^. 

The data from PARP-1 inhibition studies suggest that LPS-induced endotoxemia causes the activation of this enzyme, followed by the induction of necrotic cell death and organ damage. The cytoplasmic content, which is released after cell membrane rupture (in necrotic granulosa cells as well as in leukocytes infiltrating damaged ovarian tissue) can provoke and facilitate inflammation. We speculate that the protective action of PARP-1 inhibitor 4-HQN on LPS-induced ovarian dysfunction could also be related to the decrease in necrotic cell death. Also, anti-necrotic properties of PARP inhibitors have been shown in different animal models, including immune inflammatory pathology^[^^13^^,^^19^^,^^43^^]^. It is important that the inhibition of PARP-1 contributes to a considerable reduction in the number of cells with such *severe *DNA damage that cannot be repaired but leads to necrotic cell death. 

In conclusion, PARP-1 inhibition interrupted destructive proinflammatory connections, favored protection against genotoxic stress and led to the prevention and weakening of the pathological process. PARP-1 is an attractive target for the therapy of inflammatory disorders. However, due to the fact that this enzyme is essential for many physiological "housekeeping processes", including DNA reparation, transcription, cell cycling, mammalian oogenesis, and folliculogenesis, caution should be taken to avoid possible side effects. Our data, together with other published results, provide the ground for further studies of the underlying molecular mechanisms of cytoprotective and anti-inflammatory effects of PARP inhibitors, as well as the therapeutic potential of PARP inhibition to prevent or delay immune inflammatory diseases, including ovarian dysfunction, caused by endotoxemia.

## CONFLICT OF INTEREST

None declared.
